# Unexplained infertility as primary presentation of celiac disease, a case report and literature review

**Published:** 2011

**Authors:** Mohammadreza Ghadir, Abolfazl Iranikhah, Mahboubeh Jandaghi, Farahnaz Joukar, Massih Sedigh-Rahimabadi, Fariborz Mansour-Ghanaei

**Affiliations:** 1 Department of Gastroenterology, Qom University of Medical Sciences, Qom, Iran.; 2 Gastrointestinal and Liver Diseases Research Center, Guilan University of Medical Sciences, Rasht, Iran.

**Keywords:** *CeliacDisease*, *Infertility*, *Wightloss*, *Diarrhea*.

## Abstract

**Background: **Celiac sprue (gluten sensitive enteropathy) is an autoimmune disease which is hereditary and its pathology mainly bases on immunologic intolerance to gluten. It has a vast variety of signs and symptoms and its clinical features range from a silent disease to a typical gastrointestinal disorder. In this study we reviewed and summarized some other related issues about this disease and its relation with infertility.

**Case: **The case is a 26 years old lady who had referred to a gynecologist because of infertility for 2 years and later it revealed that she has celiac sprue.

**Conclusion:** Screening for its silent or subtle types especially among suspicious cases such as unexplained infertility seems to be a cost effective action. Meanwhile, in time administration of a gluten-free diet can lead to an almost complete cure.

## Introduction

Celiac disease (CD) known as gluten sensitive enteropathy (GSE) is an autoimmune disease which presented by hypersensitivity reaction to the gliadin of wheat and barley in genetically prone individuals and has a prevalence rate around 1% in many developed countries (1-5). Celiac has a vast variety of manifestations which range from a typical malabsorption syndrome to a subclinical iron deficiency anemia (6, 7). The clinical tabloids of CD depend on its onset time, duration and severity (8). 

Therefore, its classic presentation has an iceberg effect and each patient with CD has about 3 to 10 undetected signs or symptoms (4). Meanwhile, unlike to the traditional belief which classified CD as a digestive illness, it is a systemic disease that can occur in any time and in more than 50% of adult cases it has an atypical onset (9). There are several findings about impacts of CD on fertility among both sexes and some of these complications include; delayed menarche, menstrual discomforts, premature menopause, recurrent abortion and infertility )^10^-^11^(. Although overall prevalence of infertility is between 10- 15% and in 10- 15% of infertile women we cannot find any particular cause, CD as a risk factor of infertility and dramatic therapeutic response to gluten free diet justifying screening for this disease among unexplained infertile women (11).

## Case report

The case is a 26 years old lady who had referred to a gynecologist because of infertility 2 years ago. She has married 8 years ago and after 2 years, she decided to have child. During the first two years of marriage they used natural method for contraception and she had not used any OCP or IUD. Her menarche was at the age of 14 and from then on she has a regular and normal monthly menstruation. 

She had a negative past medical history with no sign or symptoms of neurologic diseases such as anxiety or depression. She had also no history of any clinical disease, pelvic or abdominal surgery, and extensive weight loss and drug or alcohol abuse. 

In her family history the only positive clues were; the presence of hypothyroidism, diabetes and vitiligo in her mother and aunts. In physical exams: BMI=23, secondary sexual signs were normal and there was no sign or symptom of hirsutism, acne or any other systemic disease. Evaluation of her husband revealed normal male factor and spermogram. Laboratory findings included: serum FSH, serum LH, serum prolactin, TSH, testosterone, progesterone and estradiol which all of them were at the normal range. 

Post coital test (PCT) and histrosalpangography were normal. Diagnostic laparoscopy showed no signs of tubal adhesion or endometriosis. After seven months and performing primary and secondary evaluation, she was diagnosed as a case of unexplained infertility and IVF was suggested for her. 

About 9 months ago, the patient had a medical consultation because of diarrhea and 3 kg of weight loss. She reports a history of 12 months intermittent, non bloody and osmotic diarrhea which consists of normal bowel habits intervals. There was no history of fatty stool or consumption of laxatives.

**Lab findings:**

HB: 11.8 (NL: 12-16) MCV: 76 (NL: 80-95) 

MCH: 27 (NL: 36-48) Serum Fe: 110 (NL: 80-180)

TIBC: 320 (NL: 250-460) Serum Ferritin: 54 ngr/ml (12-300) CRP: +ESR: 18 S/E: negative

OB: negative. AST: 29 IU (NL: 5-40) 

ALT: 31 IU (NL: 8-40)

Considering iron deficiency anemia, intestinal signs and the history of 4 years infertility, we became suspicious to celiac disease and checked serological factors (total IgA and tTG).

The positive results of these tests (Total IgA>40 and tTG>30) followed by upper GI endoscopy and biopsy of the second portion of duodenum. The obtained tissues were observed by two expert pathologists and they reported severe malabsorption with flattening of villi, hyperplasia of crypts and total atrophy of mucosa which was presenting stage III of modified marsh classification (Table I and Figure I). Based on patient's history and serologic and pathologic findings diagnosis of CD was confirmed and she received a complete gluten free diet. After 3 months all her clinical features including; diarrhea, flatulence and anemia were eradicated and pregnancy occurred. 

**Table I:** The modified Marsh classification

	**Type 0**	**Type 1**	**Type 2**	**Type 3a**	**Type 3b**	**Type 3c**
IEL *	<40	>40	>40	>40	>40	>40
Crypts	Normal	Normal	Hypertrophic	Hypertrophic	Hypertrophic	Hypertrophic
Villi	Normal	Normal	Normal	Mild atrophy	Marked atrophy	Absent

* Numbers are given as intraepithelial lymphocytes (IEL)/100 epithelial cells (46).

**Table II:** Common and rare signs and symptoms of Celiac disease (alphabetically ordered)*.

-Abdominal pain and discomfort (diarrhea, IBS, flatulence, borborygmus and vomiting)
-Anemia (iron, vitamin B12 and folate deficiency)
-Arthralgia and arthritis
-Bone pain (osteoporosis and osteopenia, vitamin D and calcium deficiency)
-Dermatitis herpetiform
-Elevated liver enzymes, liver failure
-Fatigue and weakness
-Growth failure in children
-Gynecologic manifestations: delayed menarche, menstrual discomforts, premature menopause, recurrent abortion and infertility.
-Neurological dysfunction (such as depression, epilepsy, migraine and ataxia)
-Vitamin and nutrients deficiency related disorders (night blindness, edema, bleeding and hematoma, peripheral neuropathy, muscle cramps, etc )
-Weight loss

* This table is driven from references (3, 13, 14-20).

**Figure 1:**
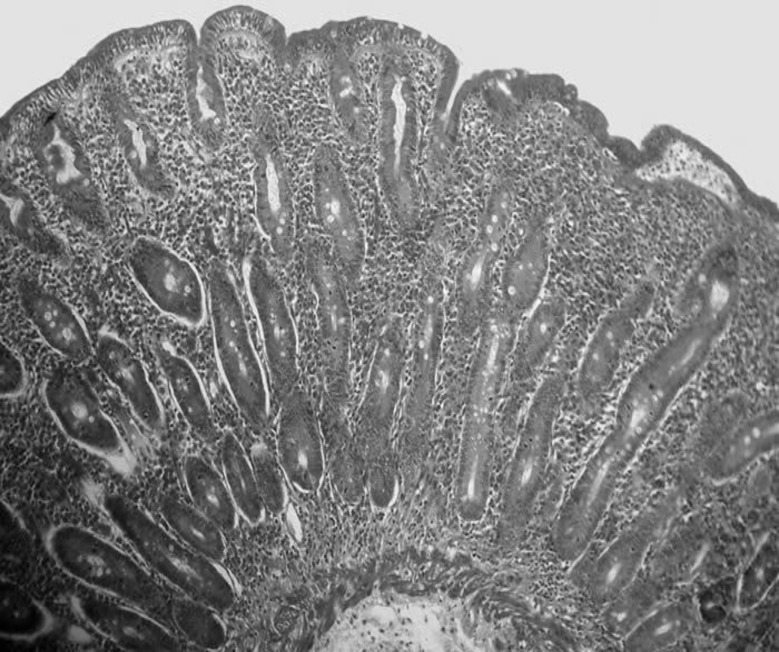
Histological view of the second portion of the patient’s duodenum

**Figure 2:**
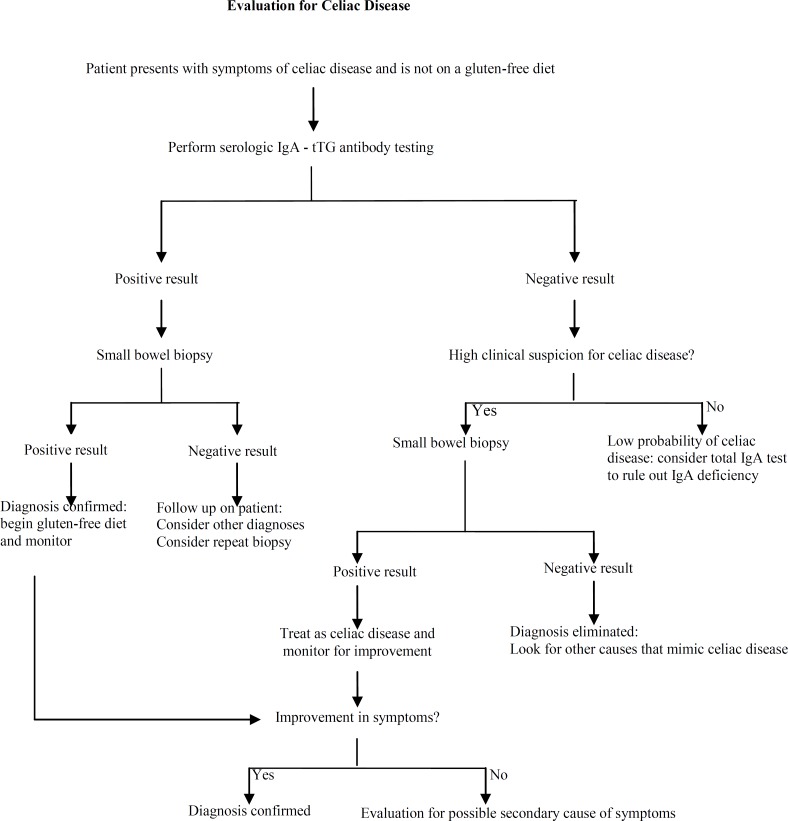
Algorithm for an approach to patients with possible celiac disease. (IgA = immunoglobulin A; tTG = tissue transglutaminase.)

## Discussion

Gluten sensitive enteropathy known as celiac disease (CD) or celiac sprue is an autoimmune disease with a prevalence of 1/270 in Finland to 1/5000 in North America (11). Manifestations of CD range from no symptoms to overt malabsorption with involvement of multiple organ systems and an increased risk of some malignancies (12). Some common and rare signs and symptoms of CD are shown in table II (3, 11, 13-20). Although CD has some classic clinical features which mainly consist of several gastrointestinal tabloids, its extra intestinal manifestations such as anemia and infertility need adequate attention and several studies put an emphasize on them. In many studies including Westerberg *et al *it is shown that CD prevalence rate is somehow more than estimations because of its silent and subtle types (21). In a study by Collin *et al* it is suggested that in unexplained infertility, silent celiac disease should be screened (22). Collin *et al* showed that the prevalence of CD among unexplained infertile cases (4.1%) is significantly higher than control group (0.0%) (p=0.02). Also, in this study it is shown that iron deficiency anemia is an important associated condition in patient with CD and unexplained infertility. Additionally, osteopenia and osteoporosis as common complications of CD (3, 23-26) put an especial importance on the time of diagnosis and treatment of suspicious cases. 

The diagnosis of CD is based on clinical suspicion plus positive serological tests and positive histological findings (11, 12, 21). The standard serologic tests which include IgA endomysial and tTG antibodies, according to many studies has a sensitivity and specificity of more than %95 (27-30). An algorithm for step by step approach to a suspicious patient with possible CD is shown in figure 2 (12). The important point is that serologic and biopsy samples should be taken before starting a gluten free diet (12, 31).

Finally all the authors believe that complete and lifelong gluten free diet is the only treatment of CD. Most of the dietary gluten is presented in wheat, rye and barely (12, 13, 21, 32-33). According to many studies, oat does not have gluten although its products are frequently contaminated with gluten (34-42). Like our case, in most of the studies, it is shown that all the complications of celiac patients can be completely cured by following a long life gluten-free diet (11-22, 43-46)

## Conclusion

Screening for silent or subtle CD especially among suspicious cases such as unexplained infertility seems to be a cost effective action and in time administration of a gluten-free diet can lead to an almost complete cure. 
